# Whispering Bloch modes

**DOI:** 10.1098/rspa.2016.0103

**Published:** 2016-07

**Authors:** B. Maling, R. V. Craster

**Affiliations:** Department of Mathematics, Imperial College London, London SW7 2AZ, UK

**Keywords:** plasmonics, asymptotics, Bloch waves

## Abstract

We investigate eigenvalue problems for the planar Helmholtz equation in open systems with a high order of rotational symmetry. The resulting solutions have similarities with the whispering gallery modes exploited in photonic micro-resonators and elsewhere, but unlike these do not necessarily require a surrounding material boundary, with confinement instead resulting from the geometry of a series of inclusions arranged in a ring. The corresponding fields exhibit angular quasi-periodicity reminiscent of Bloch waves, and hence we refer to them as whispering Bloch modes (WBMs). We show that if the geometry of the system is slightly perturbed such that the rotational symmetry is broken, modes with asymmetric field patterns can be observed, resulting in field enhancement and other potentially desirable effects. We investigate the WBMs of two specific geometries first using expansion methods and then by applying a two-scale asymptotic scheme.

## 1. Introduction

The whispering gallery phenomenon can famously be observed in the auditorium of St. Paul’s Cathedral, where a message whispered next to the surrounding wall of the circular viewing gallery can be heard clearly at any other point on the circumference, while remaining unintelligible to an observer sitting away from the wall. A mathematical explanation was provided by Lord Rayleigh in 1910 [1] and since then analogous effects, ubiquitous in wave systems with circular or spherical cavities, have been well studied [2]. In a modern setting, whispering gallery modes (WGMs) in electromagnetic wave systems have found application in spectroscopy, microdisk lasers [3], biosensors [4] and experimental testing of nonlinear optics [5]. In most instances, the excitation is partially confined due to a material boundary, and as a result the solutions are in fact quasi-modes; assuming harmonic time dependence exp⁡(−iωt) is understood, they are characterized by complex frequencies with Im(*ω*)<0, and hence decay in time as energy is radiated to infinity. A figure of merit is the so-called radiative quality factor *Q*=Re(*ω*)/|2Im(*ω*)|.

**Figure 1.**
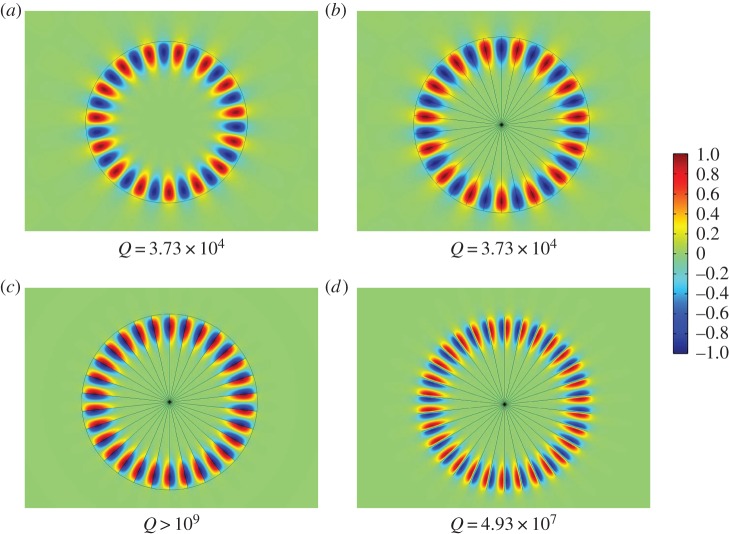
Whispering gallery modes versus whispering Bloch modes. Frame (*a*) shows a conventional WGM for a high refractive index (*n*_*r*_=2) cylinder in a low refractive index (*n*_*r*_=1) background. In (*b*), we demonstrate that the same solution exists if the cylinder is embedded with *N*=30 radial Neumann spokes. Frame (*c*) shows a WBM that exists in the latter system and is a result of the spokes themselves; a similar mode exists even if the material boundary is removed, as shown in frame (*d*). In each case, the real part of the field is plotted, normalized to the colour bar shown. Note that the theoretical *Q*-factors are far higher for the lower panels, suggesting a greater degree of confinement.

In this paper, we consider open systems with high orders of rotational symmetry. It is straightforward to prove that the quasi-modes of such systems exhibit discrete Bloch-like angular periodicity and hence we refer to them as WBMs. Unlike WGMs, these quasi-modes do not require a surrounding material boundary to achieve confinement and this can instead be induced by a geometric structure arranged periodically around a circle. To clearly distinguish WBMs from WGMs, in figure 1, we show an illustrative example displaying four apparently very similar modes. The figure depicts TE-polarized eigenstates of a high-index cylinder in vacuum, in which we place an array of spokes with Neumann boundary conditions imposed upon them (representing perfect conductors in this polarization). Irrespective of the presence of the spokes, a WGM is found due to the material mismatch between the cylinder and background, as shown in figure 1*a*,*b*. In the case with spokes, however, there is an additional mode, shown in figure 1*c*, that is created by the periodicity of the geometry; this second mode persists even if the materials are identical, as shown in figure 1*d*. We refer to the latter as a WBM. Even without the confining effect of the material boundary, in this case, the *Q*-factor is several orders of magnitude greater for the WBM than for the WGM.

In the remainder of this article, we consider cyclic arrays of inclusions embedded within a homogeneous background and therefore WGMs cannot exist. We study systems governed by the planar Helmholtz equation with Neumann boundary conditions and use two geometries to illustrate WBMs, each of which can be treated analytically using an appropriate expansion method. The first example, an arrangement of equally spaced radial spokes, is instructive as the solutions bear close resemblance to conventional WGMs as shown in figure 1. This eigenvalue problem is solved using matched eigenfunction expansions. The second example is an array of isolated circular inclusions, which is closely related to a linear array of cylinders that is well known to support Rayleigh–Bloch waves [6], but now bent into a closed curve. In this case, the methodology of multipoles is used instead. A detailed study of resonances in rings of circular inclusions was undertaken in [7], and our work is complementary to this, extending the regime of interest to larger numbers of inclusions, for which new and interesting effects are observed. More recently, similar systems have been studied as models for acoustic and electric Faraday cages; the article by Chapman *et al.* [8] focuses on shielding effects in the static and quasi-static regime, while Martin [9] develops a wire model using Foldy’s method.

Much of the motivation for this paper comes from the extensive literature on Rayleigh–Bloch surface waves on linear or planar arrays; solutions for the Helmholtz equation have been studied for comb-like structures [10,11], and also for arrays of rectangular inclusions [12], cylinders [6] and more irregular shapes [13]. General existence results have been derived for surfaces with Neumann boundary conditions [14], and for this reason, we focus our attention on these rather than the alternative Dirichlet conditions for which equivalent modes do not exist [15]. In the context of electromagnetic waves, guided modes are observed in linear or planar arrays of dielectric spheres [16] and also on conducting surfaces with periodic corrugations that support spoof surface plasmon polaritons [17,18]. It was recently shown that the quasi-modes of a grooved conducting disc give rise to magnetic localized plasmons [19]. Furthermore, there is an older literature on electromagnetic gratings showing surface waves guided by corrugations [20,21].

At a discrete set of frequencies, the angular periodicity of a system’s WBMs will match that of the rotationally symmetric structure or will be a half-integer multiple thereof. In these cases, the solutions satisfy periodic or antiperiodic boundary conditions across a single wedge-shaped repeating cell, and these are the standing wave eigenmodes of the system. Analogous standing wave solutions are found in structures with Bravais lattice periodicity [22], and this suggests that an asymptotic method based on a cyclic analogue of high-frequency homogenization [23] would be well suited to studying the systems in question. We investigate this and find, at complex frequencies close to those of the standing wave solutions, quasi-modes exist with wide-angle modulation, and we construct asymptotic expressions for the field patterns and associated complex frequencies; these modulation effects are not observed in WGM systems. We then go on to investigate the effect of slightly perturbing the geometry in a WBM system, and found that the asymptotic method can be adapted to account for this, predicting the perturbed field patterns and frequencies.

Further to the methods mentioned above, we employ finite-element method (FEM) modelling, first as a numerical check, and later in conjunction with the asymptotic theory we develop. With this approach we necessarily truncate the domain to a finite size, and particularly in the case of leakier modes with low *Q*-factors this can introduce a significant error in the calculated eigenvalues. In terms of solving the eigenvalue problem, the analytical methods are preferable as the solutions are written naturally in bases that satisfy the outgoing wave condition, and hence no such truncation is required.

We begin in §2 with the general formulation of the problem, along with a proof of the angular quasi-periodicity condition satisfied by the solutions. We then turn to the two examples that we primarily consider: the array of radial spokes and the array of isolated holes. In both cases, we use analytical methods to generate systems of equations that we solve numerically to generate discrete dispersion plots. An asymptotic method is advanced in §3 that is insightful for analysing WBMs with wide-angle modulation that occur at frequencies close to the standing wave eigenfrequencies of the system. In §4, we adapt this asymptotic method to analyse a case where the geometry of the system is slightly perturbed, leading to asymmetric field effects. Comparisons with FEM simulation are made to demonstrate the accuracy and simplicity of the approach. Finally, we draw together some concluding remarks in §5.

## 2. Prototype system

We pose an eigenvalue problem for the planar Helmholtz equation with Neumann inclusions, seeking $u:R^{2}\setminus \cup C_{j}\to C$, where $u\in C^{2}(R^{2}\setminus \cup {\bar{C}}_{j})$ and Ω∈C satisfy

2.1 $$\begin{array}{rl} & (\Delta +\Omega^{2})u(\text{x})=0,\;\text{x}\in R^{2}\setminus \cup {\bar{C}}_{j},\\ & \text{n}\cdot \nabla u(\text{x})=0,\;\;\text{x}\in \cup \partial C_{j}\\ \;\text{and}\; & \frac{\partial u}{\partial r}\to i\Omega u,\;\;r\to \infty . \end{array}\}$$

**Figure 2.**
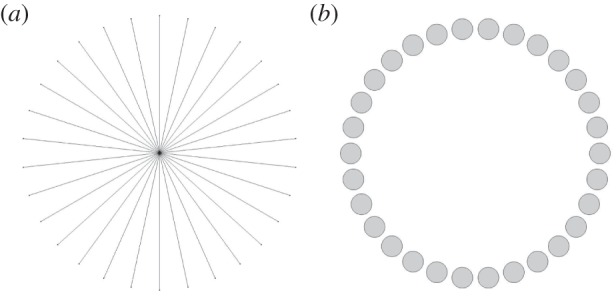
Geometries under consideration; (*a*) a system of *N* equally spaced radial spokes of unit length and infinitesimal width and (*b*) a ring of *N* circular holes of radius *a*, whose centres are equally spaced around a ring of unit radius; we take a<sin⁡(π/N) so that the holes do not overlap.

Here $C_{j}$ for $j=1,\ldots ,\tilde{N}$ refers to a set of bounded inclusions arranged to have rotational symmetry of order *N*. Note that $\tilde{N}$ may be greater or less than *N*, and a simple case to consider would be that of $\tilde{N}=N$ identical inclusions arranged periodically around a ring. The unit normal vector at an inclusion boundary is denoted by **n**, and *r*=|**x**|. In particular, we consider the two geometries shown in figure 2 so that concrete solutions can be found.

Before solving the eigenvalue problem for the two geometries under consideration, we derive a quasi-periodicity condition analogous to Bloch’s theorem for Bravais lattice structures. Consider a simple eigenvalue *Ω* of system (2.1), along the associated field *u*(*r*,*ϕ*). We introduce a discrete rotation operator $\hat{R}$ such that for any suitably defined function, $\hat{R}f(r,\phi )=f(r,\phi +2\pi /N)$. The rotational symmetry of (2.1) means that the field $\hat{R}u(r,\phi )$ solves an identical eigenvalue problem to *u*(*r*,*ϕ*), and since the eigenvalue is simple, the two must be identical up to multiplication by a complex number, i.e. $\hat{R}u(r,\phi )=\alpha u(r,\phi )$ for α∈C. If we perform the same transformation *N* times, the cyclic continuity of the problem requires that $\alpha^{N}u(r,\phi )={\hat{R}}^{N}u(r,\phi )=u(r,\phi )$ and hence *α*=e^2*mπ*i/*N*^ is one of the *N*th roots of unity. The rotational analogue of Bloch’s theorem is thus

2.2 $$u\left(r,\phi +\frac{2j\pi }{N}\right)=e^{2jm\pi i/N}u(r,\phi ),\;\forall \;j,m\in Z.$$

We do not consider the case of degenerate eigenvalues in this paper, and this possibility is left as an open question. The above result leads to a discrete Bloch-like spectrum in complex frequency space for *m*=0,1,…,*N*−1. In the examples chosen, symmetry under the transformation *ϕ*→−*ϕ* allows us to consider a reduced domain *m*=0,1,…,⌊*N*/2⌋, analogous to the irreducible Brillouin zone in conventional Bloch theory [24]. The spectra for both systems are calculated using expansion methods. We begin with the geometry of figure 2*a*.

### (a) Neumann spokes: matched eigenfunction expansion method

**Figure 3.**
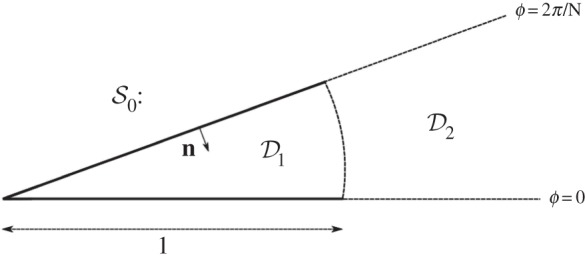
Geometry of the cell $S_{0}$. Separate expansions are used in the regions $D_{1}=\{(r,\phi ):r\in (0,1],\phi \in [-\pi /N,\pi /N]\}$ and $D_{2}=\{(r,\phi ):r\in [1,\infty ),\phi \in [-\pi /N,\pi /N]\}$.

We consider the infinite wedge-shaped repeating cell as in figure 2*a*. Supposing that one of the spokes lies on the half-line *ϕ*=0, we expand the field in the two regions shown in figure 3 using appropriate Fourier–Bessel bases. In particular, the Neumann condition must be satisfied by each term in $D_{1}$, and the quasi-periodicity condition must be satisfied by each term in $D_{2}$. The expansions are thus given by

2.3 $$u(r,\phi )=\left\{ \begin{array}{ll} \sum_{n=0}^{\infty }{\hat{A\;}\;}_{n}J_{nN/2}(\Omega r)\cos\left(\frac{nN\phi }{2}\right), & (r,\phi )\in D_{1},\\ \sum_{n=-\infty }^{\infty }{\hat{B}}_{n}H_{m+nN}(\Omega r)\;e^{i(m+nN)\phi }, & (r,\phi )\in D_{2}, \end{array}\right.$$

where *J* and *H* denote the Bessel and the Hankel functions of the first kind, respectively. To ensure that the field has the required smoothness, the expressions for *u* and its derivative ∂*u*/∂*r* from these two expansions are equated at *r*=1. We multiply the resulting equations by cos⁡(pNϕ/2) for p∈Z and integrate over the azimuthal variable *ϕ*. Using the orthogonality of the cosine functions, along with the relation

2.4 $$\begin{array}{rl} \beta_{pn} & \equiv \int_{0}^{2\pi /N}\;e^{i(m+nN)\phi }\cos\left(\frac{pN\phi }{2}\right)d\phi \\ & =\left\{ \begin{array}{ll} \frac{\pi }{N}, & m+nN=\pm \frac{pN}{2},\\ \frac{i(m+nN)(1-e^{im2\pi /N}{\left(-1\right)}^{p})}{\left(m+nN+pN/2)(m+nN-pN/2\right)}, & \text{otherwise,} \end{array}\right. \end{array}$$

we are able to eliminate the coefficients ${\hat{A\;}\;}_{n}$, leaving an infinite system of equations:

2.5 $$0=\sum_{n=-\infty }^{\infty }{\hat{P}}_{pn}{\hat{B}}_{n}\;\forall \;p\in Z,$$

where ${\hat{P}}_{pn}=\mu_{pn}^{\left(1\right)}-\mu_{pn}^{\left(2\right)}$ and

2.6 $$\mu_{pn}^{\left(1\right)}=[J_{pN/2-1}(\Omega )-J_{pN/2+1}(\Omega )]H_{m+nN}(\Omega )\beta_{pn}$$

and

2.7 $$\mu_{pn}^{\left(2\right)}=[H_{m+nN-1}(\Omega )-H_{m+nN+1}(\Omega )]J_{pN/2}(\Omega )\beta_{pn}.$$

**Figure 4.**
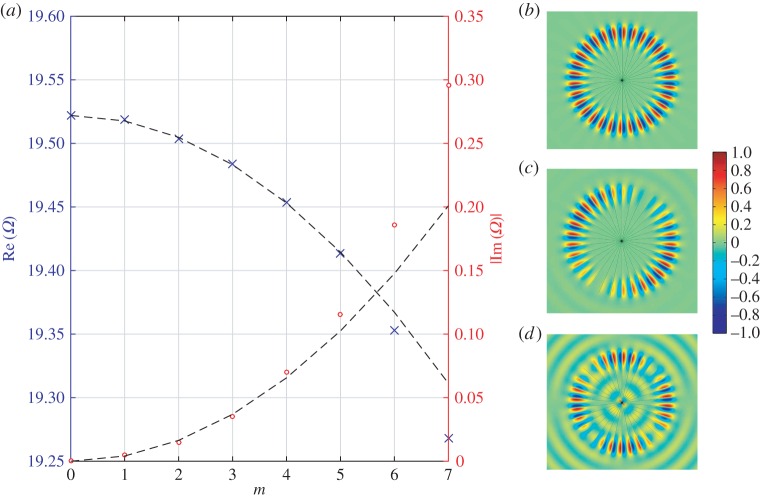
(*a*) Dispersion of the lowest branch of WBMs for a system of *N*=30 spokes. The blue crosses (red circles) show the real (imaginary) parts of the complex frequencies calculated using the method outlined in this section, and the dashed line shows the asymptotic dispersion relation (3.10) as calculated in §3. Frames (*b*–*d*) show the real part of the field *u* for modes with *m*=0,1,2, respectively, normalized to the colour bar shown. Note that the standing wave solution is periodic (*m*=0) in this case.

We note that in the study of Rayleigh–Bloch surface waves on a comb-like grating, a particularly elegant method involving residue calculus can be employed to solve the system of equations analogous to (2.5) [11,20], but unfortunately the properties of the Bessel functions in the present case prevent the same approach from being used straightforwardly here. The residue calculus method makes explicit use of the expected square-root singularity of the solution at the tip of each spoke, and a result that we can borrow readily is that the same singularity implies that ${\hat{B}}_{n}∼O(n^{-3/2})$ as n→∞. Rather than pursuing an analytical method, we truncate the infinite system (2.5) to a finite system of 2*M*+1 equations with −*M*≤*n*≤*M*, 0≤*p*≤2*M*, which we then solve numerically. A problem arises here as the higher order Bessel and Hankel functions have very small and very large magnitudes, respectively, so the resulting matrix is ill-conditioned. To remedy this, we introduce matrix elements given by $P_{pn}={\hat{P}}_{pn}/(J_{pN/2}(\Omega )H_{m+nN}(\Omega ))$, which satisfy $0=\sum_{n}P_{pn}B_{n}$ ∀ p∈Z for the same values of Ω∈C as before, but do not blow up as |*n*| or |*p*| become large. This resolves the ill-conditioning problem, but the magnitudes of the Bessel and Hankel functions themselves are still prohibitive for relatively modest values of *M*, resulting in floating-point underflow/overflow in some of the matrix elements, and failure of the scheme. In these cases, we use the asymptotic forms [25]:

2.8 $$J_{\zeta }(z)∼\frac{1}{\sqrt{2\pi \zeta }}{\left(\frac{ez}{2\zeta }\right)}^{\zeta }\;\mathrm{and}\;H_{\zeta }(z)∼-i\sqrt{\frac{2}{\pi \zeta }}{\left(\frac{ez}{2\zeta }\right)}^{-\zeta }\;\text{as }\zeta \to +\infty \text{ along the real axis},$$

along with the identity *J*_−*ζ*_(*z*)=(−1)^*ζ*^*J*_*ζ*_(*z*), and similarly for *H*_−*ζ*_(*z*), in place of the corresponding functions in *P*_*pn*_, and after simplification these are used in the truncated matrix equation **PB**=**0**, which has non-trivial solutions iff det(P)=0. For a given value of *m*, there will be a spectrum {Ω}⊂C corresponding to the quasi-modes of the open systems (figure 4), and the expansion coefficients are recovered from the null space vector **B**.

### (b) Neumann holes: multipole method

**Figure 5.**
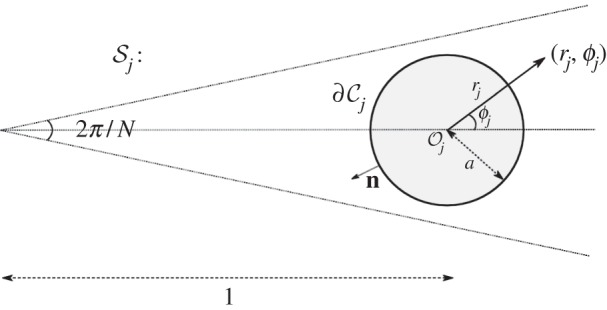
Geometry of the cell *S*_*j*_. In order for the holes $C_{j}$ and $C_{j\pm 1}$ to be non-overlapping, we require that a<sin⁡(π/N).

Other geometries are not generally well suited to eigenfunction matching, but the prototypical circular inclusion is ideally suited to multipole techniques [26,27]. Such a method was previously applied in the context of a circular ring in [7]. To solve the eigenvalue problem for the geometry of figure 2*b*, we define local systems of polar coordinates centred at each of the *j* holes (*r*_*j*_,*ϕ*_*j*_), as shown in figure 5. In the vicinity of the 0th hole $C_{0}$, the field is expanded in terms of regular and outgoing waves as

2.9 $$u(r_{0},\phi_{0})=\sum_{\mu =-\infty }^{\infty }[A_{\mu }^{0}J_{\mu }(\Omega r_{0})+E_{\mu }^{0}H_{\mu }(\Omega r_{0})]\;e^{i\mu \phi_{0}},$$

where $A_{\mu }^{0}J_{\mu }^{\prime }(\Omega a)+E_{\mu }^{0}H_{\mu }^{\prime }(\Omega a)=0$ ∀ μ∈Z ensures that the Neumann condition is satisfied on the inclusion. This expansion is valid within an annulus surrounding $C_{0}$, extending as far as the nearest point on each of the neighbouring holes $C_{1}$ and $C_{N-1}$. A second way to express the field is due to Wijngaard [28], who argued that in the absence of sources, the field existing between a number of scattering bodies must be the sum of the outgoing waves from each body. In the present case, this leads to the expansion

2.10 $$u(r_{0},\ldots ,r_{N-1},\phi_{0},\ldots ,\phi_{N-1})=\sum_{j=0}^{N-1}\sum_{\nu =-\infty }^{\infty }E_{\nu }^{j}H_{\nu }(\Omega r_{j})\;e^{i\nu \phi_{j}},$$

where the *j*th term in the first summation is given in terms of coordinates centred at the *j*th hole, and hence *u* is expressed as a function of 2*N* (dependent) variables. This expansion is valid everywhere in $R^{2}\setminus \cup C_{i}$, and automatically satisfies the outgoing radiation condition as r→∞. Quasi-periodicity implies that $E_{\nu }^{j}=E_{\nu }^{0}\;e^{2jm\pi i/N}$ ∀ ν∈Z. We use Graf’s addition theorem [29] to express every term in (2.10) in the coordinate system of the 0th hole:

2.11 $$\begin{array}{rl} u(r_{0},\phi_{0}) & =\sum_{\mu =-\infty }^{\infty }E_{\mu }^{0}H_{\mu }(\Omega r_{0})\;e^{i\mu \phi_{0}}\\ & \;-\sum_{j=1}^{N-1}\sum_{\nu =-\infty }^{\infty }A_{\nu }^{0}\frac{J_{\nu }^{\prime }(\Omega a)}{H_{\nu }^{\prime }(\Omega a)}\sum_{\mu =-\infty }^{\infty }i^{\nu -\mu }\;e^{i(2m-\mu -\nu )\pi j/N}H_{\mu -\nu }(\Omega r_{0j})J_{\mu }(\Omega r_{0})\;e^{i\mu \phi_{0}}, \end{array}$$

where *r*_0*j*_ is the distance between the centres of the 0th and *j*th holes. This expansion has the same domain of validity as (2.9), and indeed the two expressions must be equivalent. We equate the two expansions term by term, which yields, ∀ μ∈Z,

2.12 $$\sum_{\nu =-\infty }^{\infty }A_{\nu }^{0}\left[i^{\nu -\mu }\frac{J_{\nu }^{\prime }(\Omega a)}{H_{\nu }^{\prime }(\Omega a)}\sum_{j=1}^{N-1}e^{i(2m-\mu -\nu )\pi j/N}H_{\mu -\nu }(\Omega r_{0j})+\delta_{\mu \nu }\right]=0.$$

**Figure 6.**
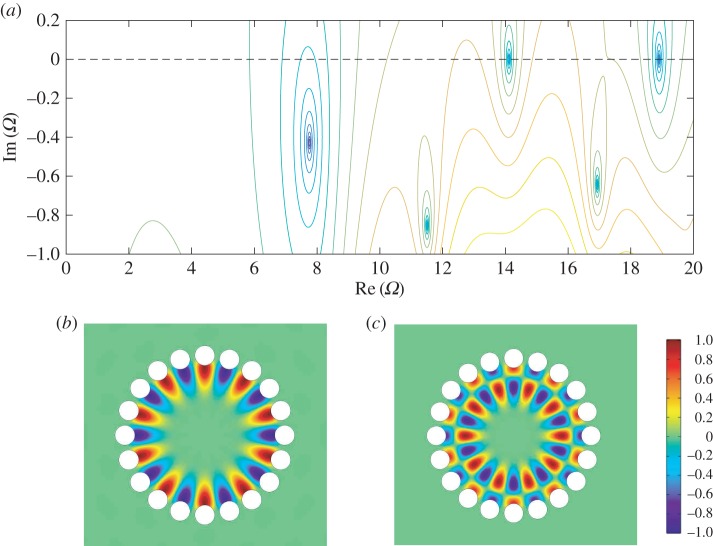
(*a*) Contour plot of log⁡|det(P)| in the complex frequency plane for a unit ring of *N*=20 Neumann holes with radius 0.8sin⁡(π/20) for standing wave solutions with azimuthal quantum number *m*=10. Note the presence of two modes very close to the real *Ω* axis, corresponding to confined quasi-modes. (*b*,*c*) The real part of the field *u* for these confined WBMs at *Ω*≈14.12 and *Ω*≈18.90, respectively, normalized to the colour bar shown.

**Figure 7.**
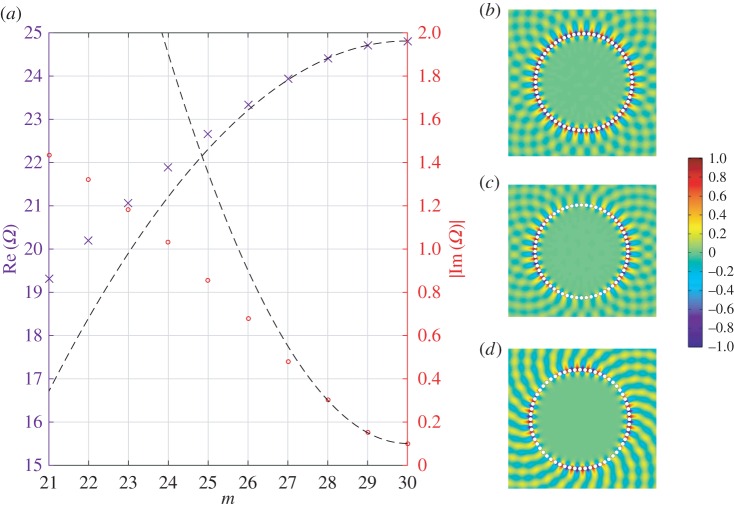
(*a*) Dispersion of the lowest branch of WBMs for a system of *N*=60 holes with radius 0.8sin⁡(π/60). The blue crosses (red circles) show the real (imaginary) parts of the complex frequencies calculated using the method outlined in this section, and the dashed line shows the asymptotic dispersion relation as calculated from (3.10) in §3. (*b*–*d*) The real part of the field *u* for modes with *m*=30,29,28, respectively, normalized to the colour bar shown. Note that the standing wave solution is antiperiodic (*m*=*N*/2) in this case.

For numerical calculation, the infinite system (2.12) is truncated to a finite system of 2*M*+1 equations with −*M*≤*μ*,*ν*≤*M*. The corresponding matrix equation **PA**=**0** has non-trivial solutions iff det(P)=0. For a given value of *m*, there will be a spectrum {Ω}⊂C corresponding to the quasi-modes of the open systems (figures 6 and 7), and the expansion coefficients are recovered from the null space vector **A**.

## 3. Asymptotic analysis

The existence of standing wave WBMs, which are periodic or antiperiodic across a single wedge-shaped elementary cell, is interesting both in terms of application and analysis. In this section, we consider large values of *N* and apply an asymptotic perturbation scheme to these standing wave solutions that allows us to calculate the field pattern, as well as the complex frequencies, of wide-angle modulated quasi-modes of the system. Later, we shall see that the same method is particularly useful in analysing cases where the geometry of the system is slightly perturbed, potentially leading to field-enhancement and other asymmetric effects.

**Figure 8.**
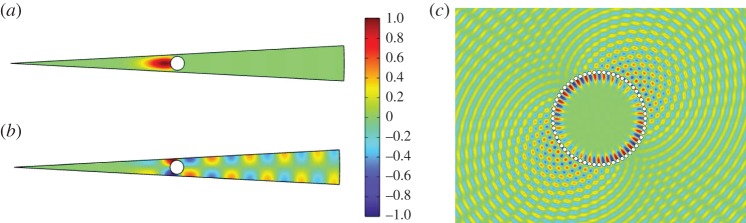
Outline of the asymptotic scheme applied to the second-lowest branch of WBMs for a ring of *N*=60 holes. (*a*) The real part of *U*_0_ for a standing wave quasi-mode with antiperiodic boundary conditions, (*b*) the corresponding real part of the function *U*_1_ and (*c*) the real part of the asymptotic field *u*_0_+*ηu*_1_ for a mode with Δ*m*=1.

The asymptotic method, outlined in figure 8, is based on high-frequency homogenization [23], which was used in the context of Rayleigh–Bloch modes on linear gratings in [10,30]. The two-scale approach relies on the disparity between the small angle subtended by the wedge-shaped elementary cell, and the large angle subtended by the underlying modulation of the quasi-mode. Motivated by this, let us consider the system (2.1), but restrict our analysis to the (arbitrary) truncated cell $S_{0}=\{(r,\phi ):r\in (0,r_{+}],\phi \in [-\pi /N,\pi /N]\}\setminus \cup C_{j}$, where *r*_+_≫1. We impose the outgoing wave condition ∂*u*/∂*r*=(*iΩ*−1/2*r*)*u* at *r*=*r*_+_, and for a sufficiently large value of *r*_+_, solutions of this problem will converge to those of the infinite problem. The standing wave WBMs satisfy periodic/antiperiodic boundary conditions on opposing sides of the cell:

3.1 $$u{|}_{\phi =\pi /N}=\pm u{|}_{\phi =-\pi /N},\;{\frac{\partial u}{\partial \phi }|}_{\phi =\pi /N}=\pm {\frac{\partial u}{\partial \phi }|}_{\phi =-\pi /N},$$

where ‘−’ is possible only if *N* is even. In order to exploit the scale disparity, we introduce a periodic angular variable satisfying *φ*∈[−*π*/*N*,*π*/*N*] in each cell, along with a slowly-varying angular variable *θ*=*ηϕ*, where *η*≪1. In the asymptotic limit *η*→0, any function of the original angular variable *ϕ* may be treated instead as a function of the two variables *φ* and *θ*, which are considered to be independent. The value of the parameter *η* is dynamic; its value is of the order of the ratio between the short-scale field variation and the long-scale Bloch modulation, but at this stage it need not be defined explicitly. The gradient and Laplacian operator are written in terms of the new variables using the chain rule as

3.2 $$\nabla \to \hat{\text{r}}\frac{\partial }{\partial r}+\frac{\hat{\phi }}{r}\left(\frac{\partial }{\partial \varphi }+\eta \frac{\partial }{\partial \theta }\right)\equiv \nabla_{r,\varphi }+\frac{\hat{\phi }}{r}\eta \frac{\partial }{\partial \theta }$$

and

3.3 $$\Delta \to \frac{1}{r}\frac{\partial }{\partial r}\left(r\frac{\partial }{\partial r}\right)+\frac{1}{r^{2}}\left(\frac{\partial^{2}}{\partial \varphi^{2}}+2\eta \frac{\partial^{2}}{\partial \varphi \partial \theta }+\eta^{2}\frac{\partial^{2}}{\partial \theta^{2}}\right)\equiv \Delta_{r,\varphi }+\frac{1}{r^{2}}\left(2\eta \frac{\partial^{2}}{\partial \varphi \partial \theta }+\eta^{2}\frac{\partial^{2}}{\partial \theta^{2}}\right).$$

Following the approach of Craster *et al.* [23], we seek solutions in the form of an asymptotic ansatz:

3.4 $$\begin{array}{rl} u(r,\varphi ,\theta ) & =u_{0}(r,\varphi ,\theta )+\eta u_{1}(r,\varphi ,\theta )+\eta^{2}u_{2}(r,\varphi ,\theta )+\cdots \\ \;\text{and}\;\Omega^{2} & =\Omega_{0}^{2}+\eta \Omega_{1}^{2}+\eta^{2}\Omega_{2}^{2}+\cdots , \end{array}\}$$

noting that the field *u* is now considered to be a function of both angular variables that are treated as independent. The resulting hierarchy is solved with periodic/antiperiodic boundary conditions on the short scale. At leading order, we have

3.5 $$O(1):\left\{ \begin{array}{ll} (\Delta_{r,\varphi }+\Omega_{0}^{2})u_{0}=0, & (r,\phi )\in S_{0},\\ \text{n}\cdot \nabla_{r,\varphi }u_{0}=0, & (r,\phi )\in \cup \partial C_{j}\cap {\bar{S}}_{0}. \end{array}\right.$$

This problem is most straightforwardly solved using an FEM solver such as Comsol [31]. Assuming the eigenvalue is simple, the solution consists of a standing wave eigenfunction *U*_0_(*r*,*φ*), multiplied by a coefficient that generally depends on the independent long-scale angular coordinate *θ*, so we have *u*_0_(*r*,*φ*,*θ*)=*f*_0_(*θ*)*U*_0_(*r*,*φ*) for an arbitrary function *f*_0_. At this point, we ensure that the value of *r*_+_ is sufficiently large that the solution has converged to that of the infinite problem (2.1), which is checked by comparing values of *Ω*_0_ with those calculated using the expansion methods in §2. Since we are interested in spatially confined quasi-modes, it is assumed that *Ω*_0_ has only a small imaginary part. Specifically, the condition that *r*_+_<1/|2Im (*Ω*_0_)| should be satisfied, which ensures the hierarchy does not break down as discussed in appendix A(b).

The next order in the hierarchy poses a forced problem:

3.6 $$O(\eta ):\left\{ \begin{array}{ll} (\Delta_{r,\varphi }+\Omega_{0}^{2})u_{1}=-\left(\frac{2}{r^{2}}\frac{\partial^{2}}{\partial \varphi \partial \theta }+\Omega_{1}^{2}\right)u_{0}, & (r,\phi )\in S_{0},\\ \text{n}\cdot \nabla_{r,\varphi }u_{1}=-\text{n}\cdot \hat{\phi }\frac{1}{r}\frac{\partial u_{0}}{\partial \theta }, & (r,\phi )\in \cup \partial C_{j}\cap {\bar{S}}_{0}. \end{array}\right.$$

At this point, we derive a compatibility condition that implies *Ω*_1_=0 (appendix A(a)). With this in place, the forced system is solved, yielding a solution of the form *u*_1_(*r*,*φ*,*θ*)=*f*_1_(*θ*)*U*_0_(*r*,*φ*)+*f*_0_′(*θ*)*U*_1_(*r*,*φ*), where *f*_1_ is another arbitrary function. The final order we consider yields another forced problem:

3.7 $$O(\eta^{2}):\left\{ \begin{array}{ll} (\Delta_{r,\varphi }+\Omega_{0}^{2})u_{2}=-\frac{2}{r^{2}}\frac{\partial^{2}u_{1}}{\partial \varphi \partial \theta }-\left(\frac{1}{r^{2}}\frac{\partial^{2}}{\partial \theta^{2}}+\Omega_{2}^{2}\right)u_{0}, & (r,\phi )\in S_{0},\\ \text{n}\cdot \nabla_{r,\varphi }u_{2}=-\text{n}\cdot \hat{\phi }\frac{1}{r}\frac{\partial u_{1}}{\partial \theta }, & (r,\phi )\in \cup \partial C_{j}\cap {\bar{S}}_{0}. \end{array}\right.$$

We derive a second compatibility condition at this order (appendix A(b)), this time yielding a second-order ODE satisfied by the modulation function *f*_0_:

3.8 $$T\frac{d^{2}f_{0}}{d\theta^{2}}+\Omega_{2}^{2}f_{0}=0,$$

where

3.9 $$T=\frac{\int_{S_{0}}(1/r^{2})(U_{0}(\partial U_{1}/\partial \varphi )-U_{1}(\partial U_{0}/\partial \varphi )+U_{0}^{2})\;dS}{\int_{S_{0}}U_{0}^{2}\;dS+\int_{-\pi /N}^{\pi /N}(i/2\Omega_{0})rU_{0}^{2}{|}_{r=r_{+}}\;d\varphi }.$$

The asymptotic solutions constructed in this section will approximate solutions to the original system (2.1) provided that the value of *r*_+_ has been chosen appropriately. A pair of linearly independent solutions to (3.8) is given by $f_{0}=\exp\left(\pm i\Omega_{2}\theta /\sqrt{T}\right)$, and the cyclic continuity of the problem demands that $\Omega_{2}^{2}/T={\left(\Delta m\right)}^{2}/\eta^{2}$ for Δm∈Z. This function governs the modulation of the periodic or antiperiodic standing wave solution; we have Δ*m*=*m* if *U*_0_ is periodic, and Δ*m*=*N*/2−*m* if *U*_0_ is antiperiodic. Using the ansatz (3.4), we derive an asymptotic expression for the complex frequencies *Ω* of the modulated quasi-modes, given the standing wave frequency *Ω*_0_:

3.10 $$\Omega =\Omega_{0}+\frac{T{\left(\Delta m\right)}^{2}}{2\Omega_{0}}+\cdots$$

This expression is valid only for the smallest values of Δ*m*=0,±1,±2,…, and ceases to be accurate when the scale-separation assumption no longer holds. For both of the systems we consider in this paper, only the most wide-angle modulated modes have *Q*-factors suggesting significant confinement, so the asymptotic method covers the regime of interest.

## 4. Geometric perturbation

The method outlined in §3 allows us to calculate asymptotic solutions to the rotationally symmetric system (2.1) that exhibit wide-angle Bloch modulation. In this section, we consider instead a case in which the geometry of the system is slightly perturbed such that this symmetry is broken. We could vary, for example, the length of or angle subtended by the spokes in the geometry of figure 2*a*, or the sizes or locations of the holes in figure 2*b*. We consider cases in which the size of the perturbation varies slowly from cell to cell; more precisely, the perturbation to the *j*th cell is proportional to a factor $g_{j}=\tilde{g}(2\pi j/N)$, where $\tilde{g}(\phi )$ is a smooth, slowly varying function of the angular variable, and hence cyclic continuity demands that *g* is periodic such that *g*_0_=*g*_*N*_. If the geometric perturbation is sufficiently small, the result of the asymptotic procedure will be a Schrödinger-type ODE in place of (3.8), and the solutions of this will provide the underlying field distribution in the perturbed system. We consider a specific example to illustrate this.

### (a) Angular perturbation in system of Neumann spokes

As a concrete example, let us consider introducing a variation in the angle subtended by each pair of adjacent spokes in the geometry of figure 2*a*. Suppose the angle between the *j*th and ( *j*+1)th spoke is given by

4.1 $$\phi_{j+1}-\phi_{j}=\frac{2\pi }{N}(1+\eta^{2}g_{j}),$$

where $g_{j}=\tilde{g}(2\pi j/N)$ for a smooth, 2*π*-periodic function $\tilde{g}(\phi )$, and $\sum_{j=0}^{N-1}g_{j}=0$. Note that, unlike in the previous section, *η*≪1 must now be defined explicitly. In terms of the separated-scale variables, $\tilde{g}(\phi )$ is considered a function of the long-scale only, and we define $g(\theta )=\tilde{g}(\phi )$. We now introduce a scaled angular variable implicitly via *ϕ*=*φ*(1+*η*^2^*g*(*θ*)), where once again *θ*=*ηϕ* is the independent long-scale angular variable. In terms of the scaled variable *φ*, each cell subtends an angle of 2*π*/*N*, so we may consider an arbitrary cell as we did in the previous section. The azimuthal partial derivative operator is expanded in two scales using the chain rule as

4.2 $$\frac{\partial }{\partial \phi }={\left(1+\eta^{2}g(\theta )\right)}^{-1}\frac{\partial }{\partial \varphi }+\eta \frac{\partial }{\partial \theta }=(1-\eta^{2}g(\theta ))\frac{\partial }{\partial \varphi }+\eta \frac{\partial }{\partial \theta }+O(\eta^{4}).$$

Having made this substitution, we seek solutions using the ansatz (3.4) as before, and find that the leading and first-order systems (3.5) and (3.6) are unchanged. The solutions *U*_0_ and *U*_1_ are carried forward to the second-order system, which is now given by

4.3 $$O(\eta^{2}):\left\{ \begin{array}{ll} (\Delta_{r,\varphi }+\Omega_{0}^{2})u_{2}=-\frac{2}{r^{2}}\frac{\partial^{2}u_{1}}{\partial \varphi \partial \theta }-\left(\frac{1}{r^{2}}\frac{\partial^{2}}{\partial \theta^{2}}-\frac{2g(\theta )}{r^{2}}\frac{\partial^{2}}{\partial \varphi^{2}}+\Omega_{2}^{2}\right)u_{0}, & (r,\varphi )\in S_{0},\\ \text{n}\cdot \nabla_{r,\varphi }u_{2}=-\text{n}\cdot \hat{\phi }\frac{1}{r}\left(\frac{\partial u_{1}}{\partial \theta }-g(\theta )\frac{\partial u_{0}}{\partial \varphi }\right), & (r,\varphi )\in \cup \partial C_{j}\cap {\bar{S}}_{0}. \end{array}\right.$$

The compatibility condition at this order is derived analogously to the one in (b), but now contains an extra term, resulting in an angular Schrödinger equation

4.4 $$T\frac{d^{2}f_{0}}{d\theta^{2}}+(\Omega_{2}^{2}-\beta g(\theta ))\;f_{0}=0,$$

where *T* is given by (3.9) and

4.5 $$\beta =\frac{\int_{S_{0}}(2/r^{2})U_{0}(d^{2}U_{0}/d\varphi^{2})\;dS}{\int_{S_{0}}U_{0}^{2}\;dS+\int_{-\pi /N}^{\pi /N}(i/2\Omega_{0})rU_{0}^{2}{|}_{r=r_{+}}\;d\varphi }.$$

**Figure 9.**
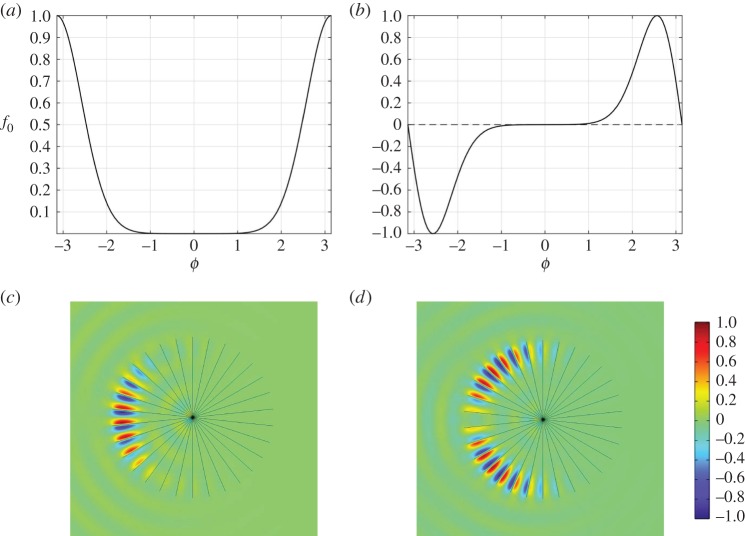
The two most confined quasi-modes of a system of 30 spokes with an angular perturbation as described in (4.1), with $\tilde{g}(\phi )=\cos(\phi )$ with *η*=2/*N*. The real parts of the modulation functions *f*_0_, calculated from equation (4.6), are shown in (*a*,*b*), with the real parts of the fields *u*, calculated by full FEM simulation, shown in (*c*,*d*).

To illustrate the accuracy of the method developed in this section, in figure 9, we show an example where a system of *N*=30 spokes is perturbed as in (4.1), with the perturbation function given by $\tilde{g}(\phi )=\cos(\phi )$, with *η*=2/*N*. In this case, the standing wave frequency is given by *Ω*_0_=19.5237, and (4.4) yields a complex Mathieu equation, given explicitly by

4.6 $$(-0.1474-0.1563i)\frac{d^{2}f_{0}}{d\theta^{2}}+(\Omega_{2}^{2}+647.36\cos(15\theta ))\;f_{0}=0.$$

Solving this using a spectral method [32], we find that the two most confined eigenvalues (those with the smallest imaginary parts) occur at $\Omega_{2}^{2}=534.61-47.28i$ and $\Omega_{2}^{2}=313.38-136.97i$. These values are substituted into the ansatz (3.4) along with the standing wave frequency to give the predicted frequencies of the modes. Table 1 shows that these calculated values are very close to those calculated using the full FEM simulation.

**Table 1.** Frequency comparison of the two quasi-modes shown in figure 9, calculated first using the asymptotic theory and then full FEM simulation.

mode from figure 9	*Ω*_Asymptotic_	*Ω*_FEM_
(c)	19.5845−0.0054i	19.5853−0.0053i
(d)	19.5594−0.0156i	19.5602−0.0155i

We note that the geometric perturbation above results in a significant reduction of the *Q*-factor compared with that of the standing wave solution in the unperturbed geometry. A similar phenomenon was observed in [7], and the asymptotic method presented here sheds light on this in the case of large numbers of inclusions: presuming that the *Q*-factor of the unperturbed standing wave is (formally) of order greater that *N*^2^, the order 1/*N*^2^ relative shift in real and imaginary parts of the frequency due to *Ω*_2_ results in the *Q*-factor being reduced to $O(N^{2})$. A detailed study of *Q*-factors in cyclic geometries will be presented in later work.

## 5. Concluding remarks

We have studied the eigenvalue problem for open systems with high degrees of rotational symmetry, governed by the planar Helmholtz equation. The WBM solutions have similarities with Rayleigh–Bloch surface waves, but fundamental difference can be identified: firstly, WBMs are quasi-modes, meaning they radiate energy to infinity and are hence characterized by complex frequencies. Secondly, the dispersion of the WBMs is discrete rather than continuous due to cyclic continuity. Thirdly, the standing wave WBMs can be either periodic or antiperiodic across one repeating cell, whereas the equivalent solutions for Rayleigh–Bloch surface waves are necessarily antiperiodic.

At frequencies close to those of the standing wave WBMs, solutions exist with wide-angle modulation, leading to dipolar and higher order radiation profiles. Analogous solutions are expected in other types of wave system, for example, those supporting in-plane elastic waves, bending waves in elastic plates and acoustic or electromagnetic waves in three dimensions. In the three-dimensional case, we expect WBMs to exist in finite structures with periodicity in the azimuthal direction. If similarly high degrees of confinement can be found for standing waves in these systems, the corresponding structures may be employed as alternatives to photonic or phononic crystal cavities in situations where confinement is desirable. Alternatively, a number of ring-like structures could be arranged on a Bravais lattice, in which case the WBM resonances could form the bases for new types of metamaterial or photonic crystal. Small perturbations of the sort considered in §4 may then be used to introduce directionality and other effects within these.

## Appendix A. Compatibility conditions in asymptotic procedure

**(a) O(η) compatibility condition**

To derive the first compatibility condition, we multiply the first line of (3.5) by the unknown field *u*_1_, and subtract the first line of (3.6) multiplied by *u*_0_. The resulting equation is integrated over the cell $S_{0}$, holding the long-scale variable *θ* constant:

A 1 $$\int_{S_{0}}(u_{1}\Delta_{r,\varphi }u_{0}-u_{0}\Delta_{r,\varphi }u_{1})\;dS=\int_{S_{0}}\left(\frac{2}{r^{2}}u_{0}\frac{\partial^{2}u_{0}}{\partial \varphi \partial \theta }+\Omega_{1}^{2}u_{0}^{2}\right)dS.$$

With the help of a Green’s identity, the left-hand side can be written as a sum of line integrals. The contributions along the opposing edges of the cell cancel by (anti-)periodicity of the *u*_*i*_’s, and after applying the boundary conditions in (3.5) and (3.6) on the inclusions, we are left with

A 2 $$\begin{array}{rl} & {\int_{-\pi /N}^{\pi /N}\left(u_{1}\frac{\partial u_{0}}{\partial r}-u_{0}\frac{\partial u_{1}}{\partial r}\right)r|}_{r=r_{+}}\;d\varphi +\int_{\cup \partial C_{j}\cap {\bar{S}}_{0}}\text{n}\cdot \hat{\phi }\frac{1}{r}u_{0}\frac{\partial u_{0}}{\partial \theta }\;dl\\ & \;=\int_{S_{0}}\left(\frac{2}{r^{2}}u_{0}\frac{\partial^{2}u_{0}}{\partial \varphi \partial \theta }+\Omega_{1}^{2}u_{0}^{2}\right)dS. \end{array}$$

We substitute in the separated-scale leading order solution *u*_0_(*r*,*φ*,*θ*)=*f*_0_(*θ*)*U*_0_(*r*,*φ*) and rearrange, which yields

A 3 $$\begin{array}{rl} & {\int_{-\pi /N}^{\pi /N}\left(u_{1}\frac{\partial U_{0}}{\partial r}-U_{0}\frac{\partial u_{1}}{\partial r}\right)r|}_{r=r_{+}}d\varphi -\Omega_{1}^{2}f_{0}\int_{S_{0}}U_{0}^{2}\;dS\\ & \;=\frac{df_{0}}{d\theta }\left(\int_{S_{0}}\frac{2}{r^{2}}U_{0}\frac{\partial U_{0}}{\partial \varphi }\;dS-\int_{\cup \partial C_{j}\cap {\bar{S}}_{0}}\text{n}\cdot \hat{\phi }\frac{1}{r}U_{0}^{2}\;dl\right). \end{array}$$

Integrating by parts, we find that the terms on the right-hand side sum to zero, so we are left with

A 4 $${\int_{-\pi /N}^{\pi /N}\left(u_{1}\frac{\partial U_{0}}{\partial r}-U_{0}\frac{\partial u_{1}}{\partial r}\right)r|}_{r=r_{+}}d\varphi -\Omega_{1}^{2}f_{0}\int_{S_{0}}U_{0}^{2}\;dS=0.$$

At *r*=*r*_+_, the field *u* satisfies the outgoing wave condition ∂*u*/∂*r*=(*iΩ*−1/2*r*)*u*. Expanding this to O(η) using the ansatz (3.4) yields

A 5 $$\frac{\partial u}{\partial r}=\left(i\Omega_{0}u_{0}-\frac{u_{0}}{2r}\right)+\eta \left(i\Omega_{0}u_{1}+i\frac{\Omega_{1}^{2}}{2\Omega_{0}}u_{0}-\frac{u_{1}}{2r}\right)+O(\eta^{2}),$$

where the terms in brackets are identified as ∂*u*_*j*_/∂*r* for *j*=0,1 respectively. Substituting these expressions for the derivatives into (A 4) yields the result

A 6 $$C\Omega_{1}^{2}\equiv \left(\int_{S_{0}}U_{0}^{2}\;dS+{\int_{-\pi /N}^{\pi /N}\frac{i}{2\Omega_{0}}rU_{0}^{2}|}_{r=r_{+}}d\varphi \right)\Omega_{1}^{2}=0.$$

For non-trivial solutions, the quantity *C* is non-zero, so we deduce that *Ω*_1_=0.

**(b) $O(\eta^{2})$ compatibility condition**

We proceed by the same method as in appendix A(a), multiplying the first line of (3.5) by the unknown field *u*_2_, and subtracting the first line of (3.7) multiplied by *u*_0_. The resulting equation is integrated over the cell $S_{0}$. We again use a Green’s identity on the left-hand side, and after applying the boundary conditions and substituting the separated-scale expressions for *u*_0_ and *u*_1_ are left with the equation

A 7 $$\begin{array}{rl} & {\int_{-\pi /N}^{\pi /N}\left(u_{2}\frac{\partial U_{0}}{\partial r}-U_{0}\frac{\partial u_{2}}{\partial r}\right)r|}_{r=r_{+}}d\varphi -\Omega_{2}^{2}f_{0}\int_{S_{0}}U_{0}^{2}\;dS\\ & \;=\frac{d^{2}f_{0}}{d\theta^{2}}\int_{S_{0}}\frac{1}{r^{2}}\left(U_{0}\frac{\partial U_{1}}{\partial \varphi }-U_{1}\frac{\partial U_{0}}{\partial \phi }+U_{0}^{2}\right)dS. \end{array}$$

Expanding the outgoing wave condition again, this time to $O(\eta^{2})$, yields

A 8 $$\frac{\partial u}{\partial r}=\left(i\Omega_{0}u_{0}-\frac{u_{0}}{2r}\right)+\eta \left(i\Omega_{0}u_{1}-\frac{u_{1}}{2r}\right)+\eta^{2}\left(i\Omega_{0}u_{2}+i\frac{\Omega_{2}^{2}}{2\Omega_{0}}u_{0}-\frac{u_{2}}{2r}\right)+O(\eta^{3})$$

and substituting these expressions for the derivatives into (A 7) yields the result

A 9 $$\begin{array}{rl} C\Omega_{2}^{2}f_{0} & \equiv \left({\int_{S_{0}}U_{0}^{2}\;dS+\int_{-\pi /N}^{\pi /N}\frac{i}{2\Omega_{0}}rU_{0}^{2}|}_{r=r_{+}}d\varphi \right)\Omega_{2}^{2}f_{0}\\ & =-\frac{d^{2}f_{0}}{d\theta^{2}}\int_{S_{0}}\frac{1}{r^{2}}\left(U_{0}\frac{\partial U_{1}}{\partial \varphi }-U_{1}\frac{\partial U_{0}}{\partial \varphi }+U_{0}^{2}\right)dS, \end{array}$$

which is rearranged to give (3.8). We stress that this equation governs the angular modulation of solutions to the truncated-cell problem. For a sufficiently large value of *r*_+_, these approximate solutions to the full planar problem (2.1), which are the ones of interest. We note, however, that if *r*_+_ is chosen to be too large, the asymptotic hierarchy will no longer hold, causing the method to fail. To understand why this is the case, we note that the converged solutions satisfy $U_{j}\propto e^{i\Omega_{0}r}/\sqrt{r}$ for *j*=0,1 as *r*→*r*_+_. Since *Ω*_0_ has a negative imaginary part, the outgoing waves grow exponentially as $r_{+}\to \infty$, causing the hierarchy to break down and the integrals in (A 9) to diverge. Since the amplitudes of these waves are minimum at *r*=1/|2 Im (*Ω*_0_)|, the hierarchy will remain intact if we choose *r*_+_<1/|2 Im (*Ω*_0_)| and this issue will be avoided.

Assuming |Im (*Ω*_0_)|≪1, there is freedom to choose *r*_+_ in the regime 1≪*r*_+_<1/|2Im (*Ω*_0_)|. It will be useful to have a bound on the error associated with making a particular choice, compared with the ‘best’ case *r*_+_=1/|2Im (*Ω*_0_)|, which may be excessively large and inconvenient for numerical methods such as FEM. Assuming the solution is sufficiently converged, the quantity C∈C appearing on the left-hand side of (A 9) is independent of *r*_+_, as shown by considering the derivative:

A 10 $$\frac{dC}{dr_{+}}={\int_{-\pi /N}^{\pi /N}\left(rU_{0}^{2}+\frac{i}{2\Omega_{0}}\frac{d}{dr}(rU_{0}^{2})\right)|}_{r=r_{+}}d\varphi .$$

Since ∂*U*_0_/∂*r*=(*iΩ*_0_−1/2*r*)*U*_0_ at *r*=*r*_+_, we have

A 11 $${\frac{d}{dr}(rU_{0}^{2})|}_{r=r_{+}}={\left(U_{0}^{2}+2rU_{0}^{2}\left(i\Omega_{0}-\frac{1}{2r}\right))\right|}_{r=r_{+}}=2i\Omega_{0}rU_{0}^{2}{|}_{r=r_{+}},$$

which is substituted into (A 10) to yield the result *dC*/*dr*_+_=0, and hence the left-hand side of (A 9) is independent of *r*_+_.

Next, we consider the right-hand side of (A 9). For converged solutions, the magnitude of the extra contribution to the integral in moving the boundary from *r*_+_ to *r*_+_′ is estimated by

A 12 |A∫r+r+′1r2(eiΩ0rr)2r dr|<maxr+<q<r+′|A1r+2∫r+q e2iΩ0r dr|<|AΩ0r+2|e2|Im(Ω0)|r+′,

where $A=\int_{-\pi /N}^{\pi /N}\;e^{-2i\Omega_{0}r}(U_{0}(\partial U_{1}/\partial \phi )-U_{1}(\partial U_{0}/\partial \phi )+U_{0}^{2})r{|}_{r=r_{+}}\;d\phi$.

Since we have also demanded that *r*_+_′<1/|2Im (*Ω*_0_)|, the variation in *T* is bounded by |*Ae*/(*CΩ*_0_*r*^2^_+_)|. A value of *r*_+_ should be chosen such that this value is significantly smaller than the higher-order terms neglected in the asymptotic expansion.
